# Role of antibodies, inflammatory markers, and echocardiographic findings in postacute cardiopulmonary symptoms after SARS-CoV-2 infection

**DOI:** 10.1172/jci.insight.157053

**Published:** 2022-05-23

**Authors:** Matthew S. Durstenfeld, Michael J. Peluso, J. Daniel Kelly, Sithu Win, Shreya Swaminathan, Danny Li, Victor M. Arechiga, Victor Zepeda, Kaiwen Sun, Shirley Shao, Christopher Hill, Mireya I. Arreguin, Scott Lu, Rebecca Hoh, Viva Tai, Ahmed Chenna, Brandon C. Yee, John W. Winslow, Christos J. Petropoulos, John Kornak, Timothy J. Henrich, Jeffrey N. Martin, Steven G. Deeks, Priscilla Y. Hsue

**Affiliations:** 1 Department of Medicine,; 2 Division of Cardiology, Zuckerberg San Francisco General Hospital and Trauma Center,; 3 Division of HIV, Infectious Diseases, and Global Medicine, Zuckerberg San Francisco General Hospital and Trauma Center,; 4 Department of Epidemiology and Biostatistics,; 5 Institute of Global Health Sciences,; 6 F.I. Proctor Foundation, and; 7 School of Medicine, UCSF, San Francisco, California, USA.; 8 Monogram Biosciences Inc., South San Francisco, California, USA.; 9 Division of Experimental Medicine, UCSF, San Francisco, California, USA.

**Keywords:** COVID-19, Cardiology, Cardiovascular disease, Cellular immune response

## Abstract

Shortness of breath, chest pain, and palpitations occur as postacute sequelae of COVID-19, but whether symptoms are associated with echocardiographic abnormalities, cardiac biomarkers, or markers of systemic inflammation remains unknown. In a cross-sectional analysis, we assessed symptoms, performed echocardiograms, and measured biomarkers among adults more than 8 weeks after confirmed SARS-CoV-2 infection. We modeled associations between symptoms and baseline characteristics, echocardiographic findings, and biomarkers using logistic regression. We enrolled 102 participants at a median of 7.2 months following COVID-19 onset; 47 individuals reported dyspnea, chest pain, or palpitations. Median age was 52 years, and 41% of participants were women. Female sex, hospitalization, IgG antibody against SARS-CoV-2 receptor binding domain, and C-reactive protein were associated with symptoms. Regarding echocardiographic findings, 4 of 47 participants (9%) with symptoms had pericardial effusions compared with 0 of 55 participants without symptoms; those with effusions had a median of 4 symptoms compared with a median of 1 symptom in those without effusions. There was no strong evidence for a relationship between symptoms and echocardiographic functional parameters or other biomarkers. Among adults more than 8 weeks after SARS-CoV-2 infection, SARS-CoV-2 RBD antibodies, markers of inflammation, and, possibly, pericardial effusions are associated with cardiopulmonary symptoms. Investigation into inflammation as a mechanism underlying postacute sequelae of COVID-19 is warranted.

## Graphical abstract

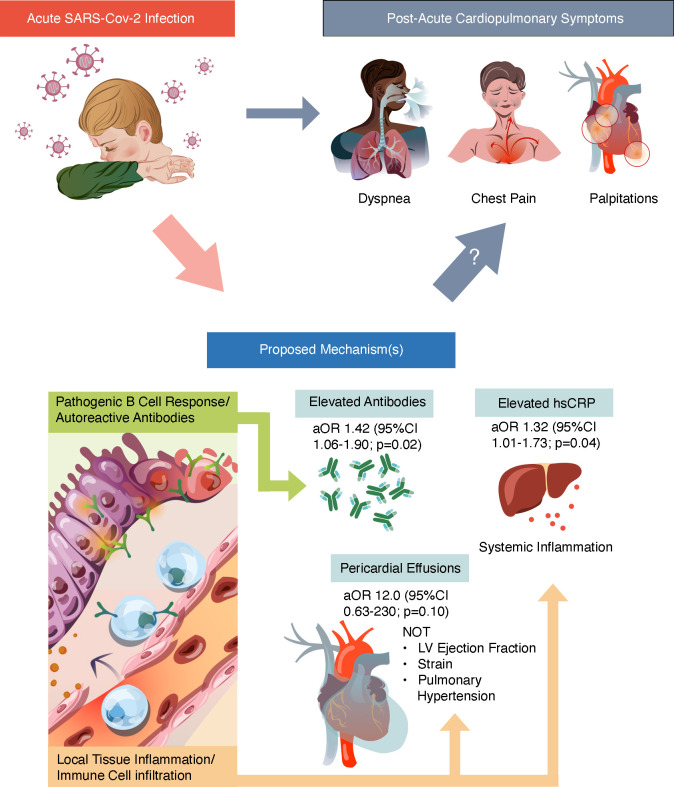

## Introduction

Following acute SARS-CoV-2 infection, a significant proportion of individuals have shortness of breath, chest pain, and palpitations (hereafter referred to as cardiopulmonary symptoms) that persist for at least 12 months (1–3). The causes of postacute cardiopulmonary symptoms are not yet known (4). Studies of hospitalized patients with COVID-19 have demonstrated that echocardiographic abnormalities are highly prevalent and associated with worse outcomes in acute COVID-19 (5–8). Yet abnormalities in cardiac function seem to resolve after hospital discharge, even among survivors with biochemical evidence of myocardial injury while hospitalized (9). In contrast, cardiac inflammation and fibrosis may be evident by cardiac magnetic resonance (CMR) imaging in some individuals 2–3 months after acute COVID-19 (10–12), but whether these changes are associated with symptoms has not been studied. Thus, whether persistent cardiopulmonary symptoms in “long COVID” or postacute sequelae of COVID-19 (PASC) are explained by cardiac structural or functional changes has become a major clinical question.

If cardiac or pulmonary pathology underlies PASC, transthoracic echocardiography (TTE) may provide clues regarding mechanisms of ongoing symptoms that may be due to either residual damage from acute infection or an ongoing cardiopulmonary process. Cardiomyopathy, for example, may be evident by abnormal systolic or diastolic function, strain, myocardial work, and evidence of elevated filling pressures. Pericarditis may be suggested by the presence of pericardial effusion. Pulmonary artery pressures can be estimated using the tricuspid regurgitant Doppler signal; pulmonary hypertension could be due to chronic thromboembolic disease, pulmonary fibrotic changes, or abnormalities in pulmonary vascular function. While resting TTE is not able to identify all possible abnormalities that could explain symptoms (such as endothelial dysfunction, for example), it is an important first tool to investigate cardiac structure and function in PASC.

To date, to our knowledge published studies have not investigated the link between persistent cardiopulmonary symptoms attributed to PASC and cardiac structural or functional changes beyond the early recovery phase (e.g., >2 months). Another major limitation of the existing literature is that most studies have included only those with severe COVID-19 requiring hospitalization, while most individuals with COVID-19, including many with persistent cardiopulmonary symptoms, were not hospitalized during acute infection. One study that examined cardiac changes 6 months after mild infection among healthcare workers using CMR had few participants with persistent symptoms and did not investigate the association between CMR abnormalities and symptoms (13).

In April 2020, a prospective cohort of individuals with asymptomatic to severe SARS-CoV-2 infection confirmed by PCR testing was established in Northern California (Long-term Impact of Infection with Novel Coronavirus [LIINC]; https://www.liincstudy.org/) (14). The objective of this study was to determine whether echocardiographic findings, cardiac biomarkers, and inflammatory biomarkers obtained months after acute COVID-19 are associated with persistent cardiopulmonary symptoms.

## Results

### Clinical characteristics among individuals with and without persistent cardiopulmonary symptoms following COVID-19.

Of the 115 people we contacted, 6 did not respond, 4 declined to participate, 2 screened out (pregnancy and congenital heart disease), and 1 participant dropped out after signing the consent but before completing a study visit. Therefore, 102 participants completed a study visit with an echocardiogram from November 2020 to May 2021, at a median of 7.2 months (IQR, 4.1–9.1 months) after SARS-CoV-2 infection defined as symptom onset or positive PCR testing among those with asymptomatic infection. As shown in Table 1, those with dyspnea, chest pain, or palpitations differed from those without those symptoms with respect to sex, BMI, and hospitalization for acute infection, with no difference in sensitivity analysis incorporating time since infection (Supplemental Table 1).

**Figure 1:**
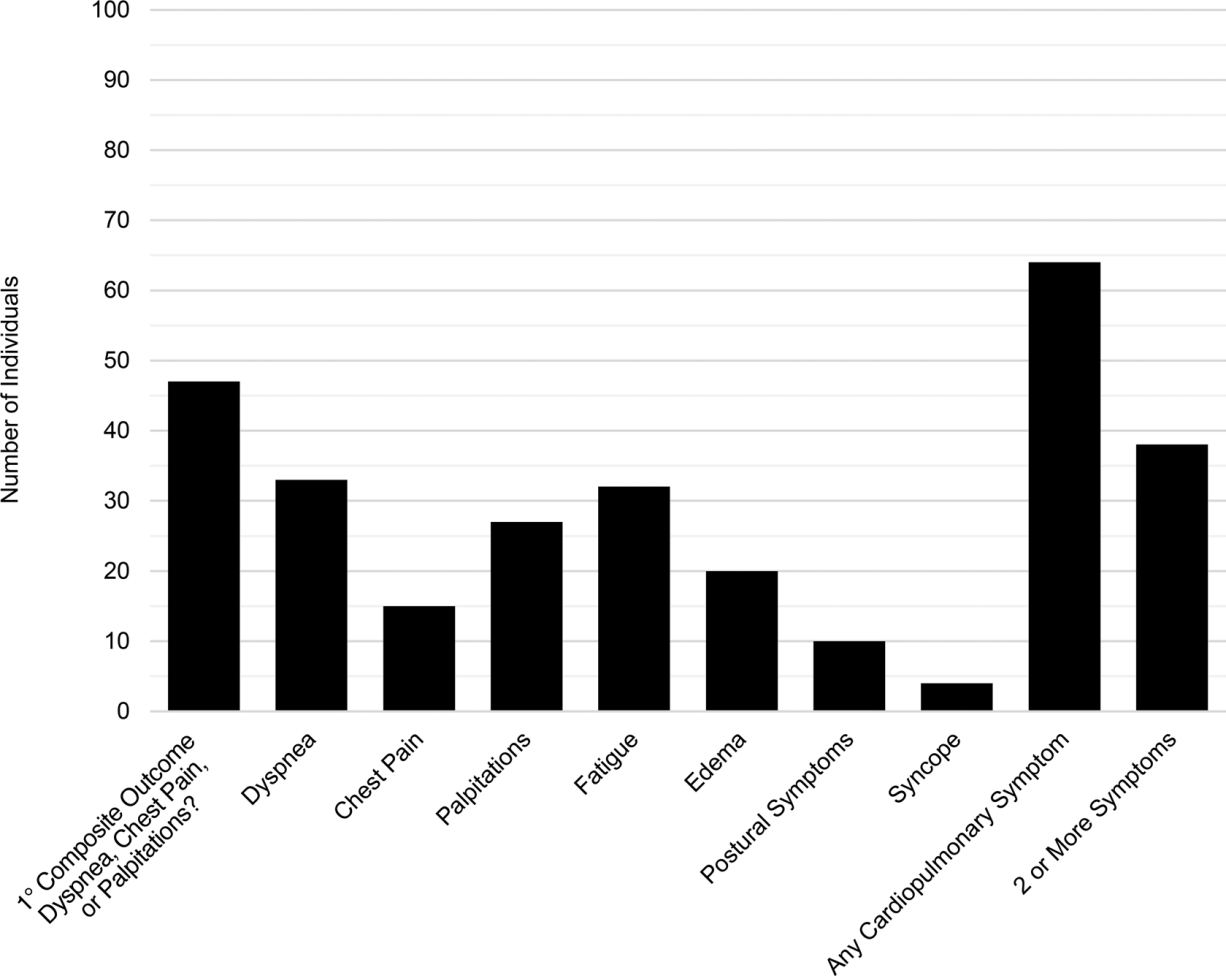
Cardiopulmonary symptoms represented in study sample. Bar plot of the number of participants who reported potentially cardiopulmonary symptoms in the 2 weeks prior to echocardiogram at a median of 7.2 months (IQR, 4.1–9.1 months) after COVID-19 onset (*n* = 102). The primary composite outcome is the presence of dyspnea, chest pain, or palpitations. These are not prevalence estimates within the population of those recovering from COVID-19, but are presented to provide context for the associations between echocardiographic findings, biomarkers, and symptoms reported here.

Of the 102 participants, 64 had at least 1 potentially cardiopulmonary symptom, including dyspnea, chest pain, palpitations, fatigue, edema, syncope, or postural symptoms. Of those, 47 individuals had the primary composite outcome of dyspnea, chest pain, or palpitations in the preceding 2 weeks: 33 had dyspnea, 15 had chest pain, and 27 had palpitations. Dyspnea (*n* = 33) and fatigue (*n* = 32) were most common (Figure 1), and 50% reported a reduction in exercise capacity. Nearly all with dyspnea characterized their shortness of breath as exertional (31 of 33, 94%), but few participants with chest pain reported association with activity (3 of 15, 20%). Palpitations were universally paroxysmal, and some participants reported sustained tachycardia after exercise or with position change. The median number of cardiopulmonary symptoms was 1 (IQR, 0–2; range, 0–6); 38 individuals reported no symptoms and 38 reported 2 or more symptoms. Symptoms remained persistent over time following acute infection (Supplemental Figure 1; supplemental material available online with this article; https://doi.org/10.1172/jci.insight.157053DS1); 14% had symptom resolution prior to their echocardiogram and 13% developed symptoms after enrolling in LIINC but before their echocardiogram.

### Sociodemographic characteristics and severity of acute illness associated with persistent symptoms.

Female sex (OR, 2.54; 95% CI, 1.13–5.74; *P* = 0.02) was associated with the composite outcome and individual symptoms, including dyspnea (OR, 2.71; 95% CI, 1.16–6.37; *P* = 0.02), chest pain (OR, 2.45; 95% CI, 0.80–7.52; *P* = 0.12), and palpitations (OR, 4.40; 95% CI, 1.70–11.4; *P* = 0.002). Hospitalization was similarly associated with the composite outcome (OR, 3.25; 95% CI, 1.08–9.82; *P* = 0.04) and with dyspnea (OR, 3.06; 95% CI, 1.04–8.78; *P* = 0.04), chest pain (OR, 1.80; 95% CI, 0.48–6.76; *P* = 0.38), and palpitations (OR, 5.45; 95% CI, 1.66-18.0; *P* = 0.005). Hypertension and HIV were associated with lower odds of symptoms. Chronic kidney disease and autoimmune disease were associated with higher odds, as shown in Table 1; the small number of individuals with specific comorbidities precludes drawing strong conclusions regarding the associations between past medical history and symptoms. There was a suggestion that oxygen therapy and admission to the intensive care unit may be associated with symptoms, but the sample of hospitalized individuals was too small for a precise estimate of these effects.

### Echocardiographic evidence of preserved cardiac function in PASC.

The prevalence of any marker of abnormal cardiac function (left ventricular [LV] ejection fraction [LVEF], <50%; diastolic dysfunction, LV strain >–18%; right ventricle [RV] strain >–20%, or qualitative RV dysfunction) was 36%, and most abnormalities were clinically minor (i.e., mild diastolic dysfunction or mildly abnormal strain). Only two individuals had LVEF less than 50%. One individual had been previously diagnosed with a dilated heart (but not clinical heart failure) attributed to HIV and was taking medical therapy for both HIV and his heart. Another individual had a myocardial infarction after infection with SARS-CoV-2, with a regional wall motion abnormality in a coronary artery distribution. Only mild diastolic dysfunction, mild RV dysfunction, mild valvular disease, as defined by ACC/AHA guidelines (15), were present, and no one had echocardiographic evidence of pulmonary hypertension.

**Figure 2:**
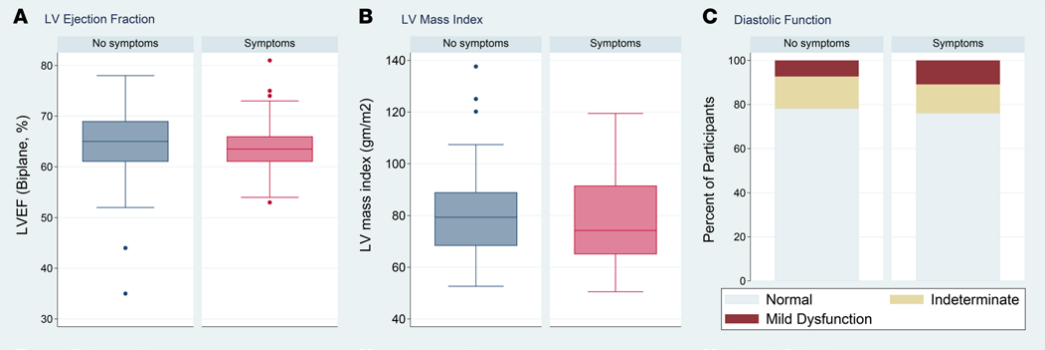
Left ventricular ejection fraction, left ventricular mass index, and diastolic function by cardiopulmonary symptoms. (**A**) Box plot of left ventricular (LV) ejection fraction (LVEF) by symptoms, (**B**) box plot of LV mass index by symptoms, and (**C**) diastolic function by symptoms (*n* = 101, 99, and 101, respectively). Odds of symptoms were 1.16 times higher per 5% decrease in LVEF, which was not statistically significant (95% CI, 0.83–1.62; *P* = 0.40). The odds of symptoms were not significantly higher with increased LV mass (1.01 per 5 g/m^2^; 95% CI, 0.89–1.16; *P* = 0.81). With regards to diastolic function, 7% of those without symptoms and 11% with symptoms had mild diastolic dysfunction; the odds of symptoms were 1.77 times higher among those with diastolic dysfunction compared with those with normal diastolic function, which was not statistically significant but could not exclude a meaningful effect (95% CI, 0.35–8.88; *P* = 0.78), especially because there were no participants with more than mild diastolic dysfunction. In **A** and **B**, boxes represent the 25th and 75th percentile, the lines inside the boxes represent medians, whiskers represent the upper and lower adjacent values (3/2 times the IQR from the end of the box) as defined by Tukey, and dots represent outliers outside the whiskers.

As shown in Table 2, other echocardiographic parameters, such as LVEF and LV mass index (Figure 2), were not clearly associated with symptoms, with two possible exceptions. Because no participants had more than mild diastolic dysfunction, our data are inconclusive regarding potential association between diastolic dysfunction and the composite outcome (OR, 1.77; 95% 0.35–8.88; *P* = 0.78). Additionally, while our data suggest that RV dilation may be associated with increased odds of symptoms, the CI is very wide, implying inconclusive results (OR, 3.55; 95% CI, 0.28–45.3; *P* = 0.31). Findings were similar when considering individual symptoms and the presence of 2 or more symptoms compared with no symptoms (Table 3) and were robust to incorporation of additional medical history (Supplemental Table 2).

Other individual functional parameters, including LV strain (OR, 1.07; 95% CI, 0.89–1.28; *P* = 0.45), RV strain (OR, 1.05; 95% CI, 0.94–1.18; *P* = 0.38), and myocardial work (OR, 1.00 per 10 mmHg percentage; 95% CI, 0.99–1.01; *P* = 0.93) were not obviously associated with the composite outcome (Supplemental Figure 2). Hemodynamic markers, including estimated cardiac index (OR, 0.91 per L/min/m^2^; 95% CI, 0.35–2.39; *P* = 0.85) and pulmonary artery pressures (OR, 1.27 per 5 mmHg; 95% CI, 0.64–2.55; *P* = 0.50), were also not substantially associated with symptoms, although a meaningful effect could not be ruled out. No participants had pulmonary hypertension (defined as a pulmonary artery systolic pressure over 35 mmHg).

### Pericardial effusions.

Among those with the composite outcome, 4 of 47 (9%) had pericardial effusions compared with 0 of 55 without symptoms (*P* = 0.038). Pericardial effusions were all trace or small, and none had echocardiographic signs of hemodynamic significance. Pericardial effusions were associated with symptoms with a large estimated OR that did not reach statistical significance (OR, 12.0; 95% CI, 0.63–230; *P* = 0.098). The median number of symptoms among those with a pericardial effusion was 4 compared with 1 among those without pericardial effusion (*P* = 0.0007; Supplemental Table 3), and pericardial effusions were associated with estimated odds of having 2 or more symptoms that were 10.7 times higher (95% CI, 0.55–206; *P* = 0.12).

None of those with pericardial effusions had heart failure, renal failure, cirrhosis, uncontrolled thyroid disease, HIV, or other autoimmune conditions that increase risk of pericardial effusions, so past medical history seems unlikely to confound this possible association between symptoms and pericardial effusions. All individuals with pericardial effusions had normal LVEF, normal strain, normal RV size and function, and normal pulmonary artery pressures; 2 had mild diastolic dysfunction. In a sensitivity analysis adjusting for autoimmune disease and HIV, the effect estimate was still large, but again, it did not reach statistical significance (OR, 9.2; 95% CI, 0.45–187; *P* = 0.15). One of the 4 individuals was diagnosed clinically with post–COVID-19 myopericarditis and treated with a nonsteroidal antiinflammatory (diclofenac) and aspirin without improvement; the other 3 had not been treated with antiinflammatory medications. Two of those with pericardial effusions had received 1 dose of the mRNA-1273 vaccine after infection but prior to their echocardiogram (1 at 2 months before and 1 at 6 months before).

### Cardiac biomarkers and hsCRP.

**Figure 3:**
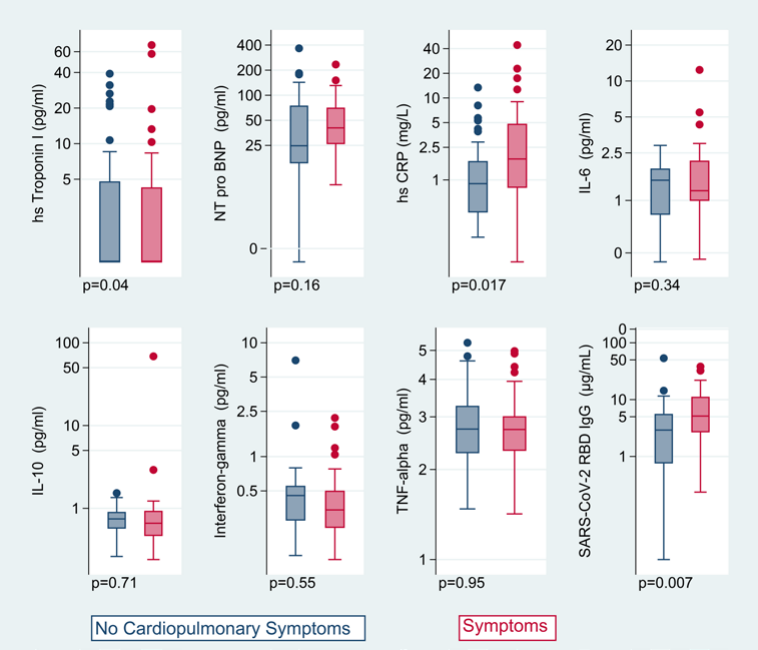
Biomarkers by presence of cardiopulmonary symptoms. Box-and-whisker plots of biomarkers plotted on log scale by presence of cardiopulmonary symptoms (no symptoms in blue on the left for each plot, symptoms in pink on the right), including hs-troponin I (*n* = 95), NT-pro-BNP (*n* = 87), hsCRP (*n* = 95), IL-6 (*n* = 73), IL-10 (*n* = 73), IFN-γ (*n* = 69), TNF-α (*n* = 73), and SARS-CoV-2 receptor binding domain IgG antibodies (*n* = 73), with *P* values listed for unadjusted *t* tests of log-transformed markers. The adjusted odds of having dyspnea, chest pain, or palpitations were 1.32 times higher per doubling of hsCRP (95% CI, 1.01–1.73; *P* = 0.02) and 1.42 times higher per doubling of antibody levels (95% CI, 1.06–1.90; *P* = 0.02). Other biomarkers were not strongly associated with symptoms. Boxes represent the 25th and 75th percentile, lines inside the boxes represent medians, whiskers represent the upper and lower adjacent values (3/2 times the IQR from the end of the box) as defined by Tukey, and dots represent outliers outside the whiskers.

Individuals with cardiopulmonary symptoms had elevated high-sensitivity C-reactive protein (hsCRP) compared with those without symptoms (median, 1.8 mg/L with symptoms vs. 0.9 mg/L without symptoms, *P* = 0.03; Figure 3 and Table 4). For each doubling of hsCRP, the odds of having the composite outcome were 1.32 times higher (95% CI, 1.01–1.73; *P* = 0.02) and the odds of having 2 or more symptoms relative to no symptoms were 1.70 times higher (95% CI, 1.11–2.61; *P* = 0.02). The relationship between hsCRP and symptoms may be stronger among those with more severe acute disease; among those hospitalized, the adjusted OR was 6.40 per doubling of hsCRP (95% CI, 1.02–40.2), and among those not hospitalized it was 1.13 (95% CI, 0.87–1.48; *P*_interaction_ = 0.068). There was no estimated substantial association between high-sensitivity troponin I (hs-troponin) and symptoms (OR, 1.02 per doubling, 0.78–1.34; *P* = 0.86), with the CI excluding a large effect. The association between N-terminal prohormone B-type natriuretic protein (NT-pro-BNP) and symptoms was inconclusive (OR, 1.27; 95% CI, 0.85–1.89; *P* = 0.25).

### Subset with SARS-CoV-2 antibody levels and additional inflammatory biomarkers.

Additional antibody and inflammatory biomarker data were available for 73 of 102 (72%) participants, mainly those enrolled early in the cohort (Figure 3). Participants with additional biomarker data did not differ from those without by age, sex, or hospitalization status, but they did have earlier dates of acute infection (median, April 2020 vs. November 2020, among those with and without biomarker data, respectively). In this subset, we observed higher SARS-CoV-2 receptor binding domain IgG antibody levels among those with symptoms compared with those without symptoms (median, 5.1 vs. 2.9 μg/mL; *P* = 0.02). The odds of having the composite outcome were 1.42 times higher per doubling of antibody levels (95% CI, 1.06–1.90; *P* = 0.02), and the odds of having 2 or more symptoms were 1.39 times higher (95% CI, 0.96–2.01; *P* = 0.09). Although the odds of having the composite outcome were not statistically significantly higher per doubling of IL-6 (OR, 1.33; 95% CI, 0.75–2.37; *P* = 0.33), the odds of having 2 or more symptoms versus no symptoms were 4.01 times higher per doubling of IL-6 (95% CI, 1.20–13.2; *P* = 0.02). Results for IL-10, IFN-γ, and TNF-α did not demonstrate any significant associations with the composite outcome (Table 4) and having 2 or more symptoms (data not shown).

### Correlations among antibody levels, hsCRP, IL-6, and pericardial effusions.

**Figure 4:**
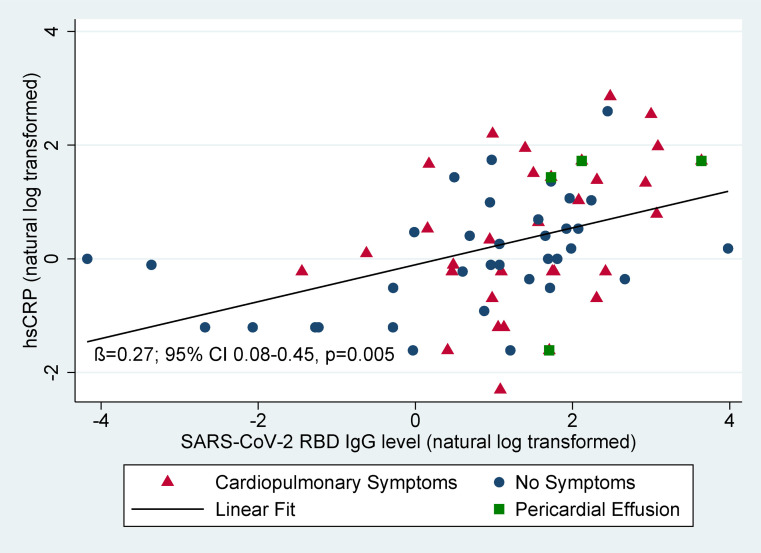
Relationship between antibodies and hsCRP. Natural log-transformed antibody levels and hsCRP are correlated (adjusted β = 0.27; 95% CI, 0.08–0.45; *P* = 0.005). Both antibody levels and hsCRP are higher in those with symptoms (red triangles) than those without symptoms (blue circles). The association between antibody levels and hsCRP did not vary by symptom status (*P* for interaction = 0.51 and minimal change in β coefficient). Those with pericardial effusions (green squares) had higher antibody levels and higher hsCRP.

The odds of having a pericardial effusion were estimated to be 1.98 times higher per doubling of IL-6 (95% CI, 0.85–4.63; *P* = 0.12), 1.35 times higher per doubling of hsCRP (95% CI, 0.75–2.43; *P* = 0.32), and 1.87 times higher per doubling of SARS-CoV-2 antibody levels (95% CI, 0.92–3.81; *P* = 0.09), though none of these results reached statistical significance. No individuals with pericardial effusions had a high-sensitivity troponin levels of more than 5 pg/mL at the time of the echocardiogram suggestive of ongoing myocarditis. Biomarkers among those with and without pericardial effusions are shown in Supplemental Figure 3. We found a statistically significant linear correlation between log-transformed antibody levels and hsCRP (adjusted β = 0.27; 95% CI, 0.08–0.45; *P* = 0.005; Figure 4). The association between antibody levels and hsCRP did not vary by symptom status (*P* for interaction = 0.51 and minimal change in β coefficient). Antibody levels were correlated with IL-6 levels only among those with symptoms (β = 0.25; 95% CI, 0.001–0.51; *P* = 0.05; Supplemental Figure 4).

### Role of acute treatments and postinfection vaccines.

The median date of infection was June 2020 (IQR, March 2020 to September 2020), and only 19 of 102 participants (19%) were hospitalized. Therefore, few participants received acute treatment for COVID-19. Only 3 participants were treated with remdesivir (with 1 additional blinded to remdesivir vs. placebo; all hospitalized), 3 were treated with hydroxychloroquine, 11 were treated with azithromycin (6 hospitalized), and 5 with corticosteroids (3 hospitalized). In exploratory analyses given this small number of treated participants and high risk for confounding by indication, none of these treatments were significantly associated with the primary outcome (remdesivir, *P* = 0.77; steroids, *P* = 0.19; azithromycin, *P* = 0.38).

Among the study sample, 82 of 102 participants (80%) had not been vaccinated at the time of their echocardiogram, and 20 participants (20%) had received 1 dose of vaccine with a median time from vaccine to echocardiogram of 34 days (IQR, 17–53 days). There was no difference in the prevalence of the primary outcome by vaccination status (38 of 82 participants, 46% among unvaccinated; 9 of 20 participants, 45% among vaccinated; *P* = 0.91). The odds of symptoms remained 1.34 times per doubling of hsCRP (95% CI, 1.03–1.76, *P* = 0.035), accounting for vaccine status, and there was not a significant interaction between vaccine status and hsCRP on symptoms (*P*_interaction_ = 0.35). Similarly, the odds of symptoms were 1.47 higher per doubling of antibody levels (95% CI, 1.12–1.95; *P* = 0.006) without a statistically significant interaction (*P*_interaction_ = 0.69). There were no significant changes in the lack of association between other echocardiographic parameters and symptoms when incorporating vaccine status, with the possible exception of pericardial effusion. Vaccination after COVID-19 was associated with 4.54 times higher odds of a pericardial effusion (95% CI, 0.73–28.3; *P* = 0.11), as compared with not being vaccinated at the time of the echocardiogram**,** although this was not statistically significant. There was not a statistically significant interaction between pericardial effusions and vaccine status on symptoms (*P*_interaction_ = 0.88), with an estimated OR for symptoms among those with pericardial effusions of 6.27 (95% CI, 0.29–135; *P* = 0.24) among the unvaccinated and 8.85 (95% CI, 0.37–214; *P* = 0.18) among the vaccinated.

## Discussion

In this cross-sectional analysis of a prospective COVID-19 recovery cohort, we found that higher antibody levels, markers of inflammation (hsCRP and possibly IL-6), and possibly pericardial effusions were associated with cardiopulmonary PASC at a median of 7 months after infection with SARS-CoV-2. Taken together, these data suggest that symptoms in cardiopulmonary PASC are not due to myocardial injury or changes in cardiac function, but rather associated with higher antibody levels and a systemic inflammatory process. To our knowledge this is the first study to demonstrate a possible association between the presence of pericardial effusions and inflammatory biomarkers in the setting of PASC. Our findings are an important step in understanding the mechanism of PASC that will be critical to treat and prevent this complication, which affects a meaningful proportion of individuals following SARS-CoV-2 infection.

### Determinants for cardiopulmonary PASC.

The two patient characteristics strongly associated with ongoing cardiopulmonary symptoms were female sex and hospitalization for acute COVID-19. Female sex has been previously associated with COVID-19 symptoms lasting greater than 28 days (16), 6 months (17), and 1 year (3). Several studies have previously demonstrated that severity of initial illness and, specifically hospitalization, are associated with symptoms at 30 and 60 days and at 6 months (17, 18). Few of our participants were treated with evidence-based therapies for acute COVID-19, as they had not yet been identified early in the pandemic, so we were not able to examine the effect of acute treatment on development of PASC.

### Antibody levels and inflammation in cardiopulmonary PASC.

We demonstrated an association between higher antibody levels and elevated markers of inflammation, namely hsCRP, with cardiopulmonary symptoms, which supports the role of inflammation as a putative mechanism underlying cardiopulmonary PASC rather than myocardial injury resulting in abnormal cardiac function. Our findings are consistent with prior studies that found higher antibody levels among those with symptoms at 6 months (17, 19), although a separate study found lower antibody levels but higher CRP among those with the most symptoms (20). Our study is also consistent with a prior study that found normal hs-troponin and NT-pro-BNP levels among individuals 6 months after mild infection compared with matched uninfected controls (13).

Higher levels of antibodies among those with symptoms could reflect more severe initial infection (19) or possibly viral antigen persistence (21, 22), both of which have been suggested as contributors to PASC; either process could be related to persistent inflammation we found. We did not examine viral persistence in vivo as part of this study. The identification of pericardial effusions among a subset of individuals with symptoms and elevated antibody levels, hsCRP, and IL-6 among those with pericardial effusions raises the possibility that localized organ inflammation may be present beyond the early convalescent phase (2–3 months) where pericardial effusions have been noted in CMR studies (11, 12, 23, 24). Troponin was low or undetectable among all those with pericardial effusions, providing strong evidence against ongoing myocardial injury or active myopericarditis. Whether or not this phenomenon is distinct from postviral pericarditis (25), well-described after other viruses, is uncertain. We also cannot exclude an association between postinfection vaccination with the mRNA-1273 vaccine and subsequent pericardial effusions. The presence of pericardial effusions following COVID-19 is similar to pericardial effusion in the setting of HIV before the antiretroviral era (26).

### Lack of evidence supporting cardiac structural or functional pathology underlying PASC.

Half of those with cardiopulmonary symptoms attributed to COVID-19 had no structural or functional abnormalities evident on echocardiography, and the functional abnormalities present among the remainder did not provide important clues to the etiology of symptoms, with the possible exceptions of diastolic function and RV dilation. Our findings provide strong evidence that active ongoing myocarditis leading to heart failure or persistent pulmonary hypertension from residual lung injury are not common pathways to symptoms among the majority of those with cardiopulmonary PASC. Other organic cardiac pathologic changes that may underlie cardiopulmonary PASC that are not detectable by resting echocardiography include ischemia, including that due to microvascular/endothelial dysfunction (12, 27), abnormal diastolic function, or pulmonary hypertension in the setting of exertion (28), and autonomic dysfunction due to nerve involvement (29, 30).

Our study is consistent with previously published echocardiographic studies in early COVID-19 convalescence. For example, several studies found a high prevalence of persistent cardiopulmonary symptoms lasting up to 1–3 months after hospitalization for acute infection, but a low prevalence of echocardiographic abnormalities and normal LV and RV function (31–33). Two studies performed TTE 6 months after hospitalization for COVID-19 in a total of 94 individuals and found no abnormalities on TTE performed at rest and no differences between those with and without myocardial injury during acute infection (28, 34). Our study extends upon these findings by including individuals not hospitalized for severe COVID-19, demonstrating that significant cardiac functional changes are not common more than 6 months following acute SARS-CoV-2 infection and including assessment of antibody levels and inflammatory markers.

Our findings are consistent with those of CMR structural and functional studies, which demonstrated normal LV and RV function as the predominant findings (10, 12, 23). Although several studies demonstrated evidence of inflammation and late gadolinium enhancement suggestive of fibrosis in a high proportion of participants early after acute SARS-CoV-2 recovery, the long-term implications of these findings remain uncertain and will require further investigation. Other studies have suggested that myocarditis and cardiac fibrosis after COVID-19 may be rare, with a prevalence of less than 1% among young healthy athletes (35, 36) and no difference between healthcare workers with mild SARS-CoV-2 infection and controls (13).

Acute SARS-CoV-2 infection can cause pneumonia and acute respiratory distress syndrome. PASC may be related to residual or ongoing pulmonary damage or dysfunction, but we did not assess pulmonary structure or function in this study. Others have demonstrated that reduced diffusion capacity on pulmonary function testing and residual ground glass opacities or fibrotic changes on chest computed tomography at 6 months and 12 months are common among hospitalized survivors and highly associated with severity of acute infection (37–39). The prevalence of these abnormalities among nonhospitalized people with PASC and their association with symptoms is not well established. None of our participants had pulmonary hypertension, so chronic thromboembolic disease (which typically presents as pulmonary hypertension) is unlikely to explain symptoms in our sample. It is possible that changes in pulmonary architecture or function may explain at least some of the symptoms in our cohort.

Another potential mechanism for cardiopulmonary symptoms in PASC that was not investigated is endothelial or microvascular dysfunction (40–42). Endothelial cells express SARS-CoV-2 targets for cell entry, angiotensin-converting enzyme 2 (ACE2) receptor, and transmembrane serine protease 2 (TMPRSS2), allowing for direct viral infection of endothelial cells (43). Microvascular dysfunction and thromboembolism have been implicated in the pathology of acute COVID-19 (41, 42), with autopsy studies demonstrating endotheliitis (44). Two studies have demonstrated abnormal endothelial function at 3 months after acute COVID-19 (27, 45). Vascular inflammation may be evident using 2-deoxy-2-[^18^F]fluoro-D-glucose positron emission tomography among those with PASC (46); similarly, reduction in myocardial perfusion reserve suggestive of coronary microvascular dysfunction has been reported in PASC (47). Additional investigation is needed to understand whether endothelial and/or microvascular dysfunction are associated with symptoms in PASC (48).

### Therapeutic implications — cardiac evaluation and antiinflammatory therapy for PASC.

Given the millions of individuals who have been infected with COVID-19 globally, and emerging reports of persistent symptoms in a meaningful proportion of individuals (37, 49–52), studies to identify and ultimately treat or prevent PASC are of the utmost importance. Clinical evaluation and management for individuals with suspected cardiopulmonary PASC are unknown but could include biomarker measurement, electrocardiogram, and echocardiogram and should be tailored to each patient’s clinical scenario, particularly to rule out non–COVID-19 pathology and to target consideration for advanced testing, such as CMR or cardiopulmonary exercise testing.

With regards to therapeutic strategies, antiinflammatory approaches have had differing results in the setting of acute COVID-19. Dexamethasone reduces mortality only among critically ill individuals with COVID-19 (53, 54). JAK inhibitors (ruxolitinib and baricitinib) reduce use of mechanical ventilation and mortality among adults hospitalized with severe COVID-19 (55). Trials of IL-6 inhibitors (tocilizumab and sarilumab) have had mixed results (56–62), but meta-analysis suggests that tocilizumab may reduce 28-day mortality (63). Colchicine had no effect on mortality among hospitalized patients (64, 65), and findings among community treated patients were not statistically significant except in among those with PCR-confirmed infection (66). None of these antiinflammatory or immunomodulatory therapeutic strategies have been evaluated for PASC, nor have any of these trials reported long-term follow-up to evaluate whether acute treatment with antiinflammatory therapy reduces risk of PASC. Therefore, mechanistic studies with long-term follow-up are needed to elucidate the role of inflammation in PASC during both the acute and recovery phases. Given our findings, investigation of antiinflammatory therapies to alleviate or prevent persistent cardiopulmonary symptoms following COVID-19 may be reasonable to consider but will require investigation in future clinical trials.

### Study limitations.

Limitations of this study include the use of a convenience sample and the cross-sectional echocardiographic and biomarker assessments. As with any observational study, there is a risk for residual confounding, including unmeasured confounding; we have tried to minimize the magnitude of these effects by conducting additional sensitivity analyses adjusting for additional possible confounders with minimal changes to our results (Supplemental Table 2). There is a risk of selection bias from those in the LIINC study who chose to participate in the cardiovascular substudy and from the shift in our recruitment criteria toward those with symptoms; however, the prevalence of the primary composite outcome and our findings were no different in post hoc analysis including only those enrolled without respect to symptoms (Supplemental Table 4). Selection bias might increase the prevalence of echocardiographic abnormalities compared with the target population, which we expect would bias our results toward finding abnormalities among those with symptoms.

To date, there are no formal definitions of cardiopulmonary PASC; an overly sensitive definition of PASC could bias our results toward the null, which was our rationale for not including those only reporting fatigue or edema without other cardiopulmonary symptoms in the primary composite outcome. As we did not query people prior to infection with SARS-CoV-2, recall bias could influence perception of new symptoms. We did not have echocardiograms from before or during acute infection to examine subclinical changes among our sample or to establish whether findings (such as diastolic dysfunction, for example) were present prior to infection with SARS-CoV-2. It is possible that COVID-19 exacerbated preexisting subclinical cardiovascular disease that preceded infection and is associated with symptoms. Because we did not include pulmonary measures (chest computed tomography and pulmonary function tests, for example) or measures of endothelial or microvascular function, we were unable to assess if symptoms were due to primary pulmonary or vascular pathology.

Because we are specifically interested in the pathophysiology in those with persistent symptoms compared with individuals who fully recovered from COVID-19, we intentionally did not include a SARS-CoV-2 uninfected control group. We acknowledge that inclusion of such a control group would have strengthened our inferences, particularly with respect to pericardial effusions and biomarkers. We excluded those with preexisting heart failure, congenital heart disease, and pulmonary hypertension, so our findings may not be generalizable to those with preexisting cardiac disease. Third, because we only included a small number of people who received intensive care during acute COVID-19 and none with myocarditis in the setting of acute disease, our findings may not be applicable to those with the highest severity of illness. Finally, the number of participants with pericardial effusions was small, so findings, particularly with respect to biomarkers and pericardial effusions, should be confirmed in larger studies.

### Conclusions.

In conclusion, SARS-CoV-2 antibody levels, inflammatory biomarkers, and possibly pericardial effusions were associated with cardiopulmonary symptoms, but other echocardiographic structural and functional parameters were not associated with PASC (with possible exceptions of diastolic dysfunction and RV dilation, which were inconclusive). Further studies using advanced cardiopulmonary testing, including CMR and cardiopulmonary exercise testing, and into mechanisms of elevated antibody levels and increased inflammation in PASC will provide much needed insight into therapeutic targets in this disease process.

## Methods

### Design

We performed cross-sectional evaluation of a prospective cohort study.

### Participants

As previously described, the LIINC COVID-19 recovery cohort (NCT04362150) was established in April 2020 to study the effects of infection with SARS-CoV-2 (67). Most participants were recruited from the general community, with some referred from acute studies. Confirmed SARS-CoV-2 infection with documentation of nucleic acid amplification testing was required. We defined the date of infection based on symptom onset if symptoms preceded positive PCR testing or as the date of the positive PCR in presymptomatic or asymptomatic individuals. Individuals were queried regarding the presence of 32 individual symptoms from the Centers for Disease Control list of COVID-19 symptoms and from the Patient Health Questionnaire Somatic Symptom Scale (68). A symptom was considered present if it was new in onset or worsened (if preexisting) since the time of SARS-CoV-2 infection. We invited the first 95 participants who sequentially indicated interest in participating without respect to symptom status and enrolled all eligible and willing individuals (*n* = 78); thereafter, we continued to enroll all eligible people living with HIV (*n* = 13) and selectively enrolled those without HIV who reported symptoms at their prior LIINC study visit (*n* = 11). We excluded pregnant individuals (due to normal dynamic changes in cardiac function throughout pregnancy that could potentially confound our results) and those with history of heart failure, pulmonary hypertension, moderate or severe valvular disease, congenital heart disease, or organ transplant prior to COVID-19 first through self-report by participants; those who reported heart disease had available clinical records reviewed for medical history, medications, and surgical history to determine eligibility.

### Measurements

#### Questionnaire based.

Participants completed a structured interview concerning medical history prior to SARS-CoV-2 infection, characteristics of acute infection, cardiopulmonary diagnoses, and symptoms within the previous 2 weeks, which were considered potentially COVID-19 related only if new or worsened. We systematically asked about fatigue, shortness of breath, chest pain, palpitations, syncope, and edema. After reports of postural orthostatic tachycardia syndrome we added questions about positional symptoms (29). Demographics including sex, race, ethnicity, income, and education were self-reported by participants.

#### Echocardiographic.

We measured supine blood pressure. A cardiac sonographer blinded to the clinical characteristics and symptom status of participants performed echocardiograms using a standardized protocol with a GE VIVID E90 machine. Echocardiograms were measured and postprocessed by a single echocardiographer with GE EchoPAC software. Volumes and ejection fraction were measured using the modified Simpson’s rule using the biplane method of discs and indexed to body surface area (69). Diastolic function was assessed according to the 2016 American Society of Echocardiography guidelines (70). Additional quantitative parameters were measured, including early-to-late diastolic filling ratio (E/A), early diastolic lateral and medial mitral annulus tissue Doppler velocity (e′), early diastolic filling to tissue velocity ratio (E/e′), tricuspid annular plane systolic excursion, and RV systolic excursion velocity (S′). Pulmonary artery pressures were estimated based on tricuspid regurgitant continuous-wave Doppler tracings using the modified Bernoulli equation and right atrial pressure estimated by inferior vena cava size and collapsibility. Cardiac index was estimated using LV outflow tract velocity time integrals and diameter indexed to body surface area. LV peak systolic global longitudinal strain, peak systolic dispersion, and RV free wall peak systolic strain were measured using focused ventricular views at greater than 60 frames per second. Myocardial work, constructive work, wasted work, and work efficiency were measured based on strain measurements and noninvasive blood pressure. Presence of pericardial effusion was assessed from multiple views and confirmed by a second echocardiographer blinded to all other participant data.

#### Blood based.

Two separate blood specimens were evaluated in this study. All participants had venous blood collected on the day of the echocardiogram that was processed for serum and plasma. Samples were batch processed for measurement of hs-troponin (ADVIA Centaur High-Sensitivity Troponin I [TNIH] assay), hsCRP (ADVIA Chemistry CardioPhase High Sensitivity C‑Reactive Protein assay), and NT-pro-BNP (Roche Cobas 6000 Elecsys pro-BNP II assay). A subset of participants (*n* = 73) had antibodies and additional markers already measured through the LIINC study utilizing blood collected 90–160 days after symptom onset analyzed by Monogram Biosciences using the Quanterix Simoa platform with Simoa Assay Kits provided by Jeremy Lambert from Quanterix (Billerica, Massachusetts, USA) free of charge. These included SARS-CoV-2 receptor binding domain IgG, IL-6, IL-10, IFN-γ, and TNF-α. Samples were assayed blinded with respect to patient and clinical information. Assay performance was consistent with the manufacturer’s specifications.

### Statistics

As our primary outcome of interest was the presence of cardiopulmonary PASC, our primary analyses consisted of logistic regression models first for the composite outcome of presence of dyspnea, chest pain, or palpitations, and then for presence of each individual symptom. We classified patients based on reported symptoms for the 2-week period prior to their echocardiogram; those who had previously reported symptoms that were not present during the 2 weeks prior to the echocardiogram were not counted as having symptoms; similarly, those who previously reported no symptoms but then reported new symptoms at their study visit (i.e., in the 2 weeks prior to the echocardiogram) were counted as having symptoms. As a proxy for more severe PASC, we assessed the distribution of the number of symptoms reported to compare the tertile with the most symptoms with the tertile with the fewest symptoms; therefore, we modeled having 2 or more symptoms relative to no symptoms (71). We modeled associations between presence of cardiopulmonary symptoms (outcome) and demographic/clinical variables, including race and ethnicity, educational attainment, income, BMI, past medical history, and acute COVID-19 hospitalization as a marker of initial severity (exposures) and included age and sex as confounders. We used the same approach to model associations between symptoms and echocardiographic parameters, including age, sex, hospitalization, and time since symptom onset as confounders. Given our small sample size, we only included pertinent past medical history in adjusted models if hypothesized a priori to be a potential confounder, including hypertension for LV mass index, left atrial volume, and diastolic dysfunction and lung disease for RV parameters. Similarly, we included HIV as a covariate when supported by prior literature and assessed for interactions between HIV and LV mass index (72), diastolic function (72, 73), LV strain (74, 75), and pulmonary artery pressures (76). We conducted additional sensitivity analyses, including all past medical history.

We followed the same approach to model the association between symptoms and biomarkers, which were natural log transformed due to right-skewed distributions. We assessed for interactions between sex and troponin and NT-pro-BNP and interactions between HIV and inflammatory markers (hsCRP, IL-6, etc.). To model associations between symptoms and echocardiographic parameters where there was complete separation of the variable of interest in the fitted logistic regression model (pericardial effusions and RV function), we used Firth logistic regression (77). To model correlation between antibodies and inflammatory markers, we used linear regression of log-transformed biomarkers.

We had complete data for all demographic, past medical history, and acute COVID-19 hospitalization/severity variables except for income, for which we report a “missing/prefer not to answer” category; echocardiographic and biomarker parameters were analyzed using complete cases only. Only 2 echocardiographic parameters had a significant proportion of missing data, both of which were expected due to technical limitations: tricuspid regurgitation peak velocity used to estimate the pulmonary artery systolic pressure and RV free wall strain; for these, the numbers used for analysis are noted in the legend for Table 2. For biomarkers, we also used complete cases, with notable missingness, in that only 73 of the 102 participants had samples collected for additional biomarker and antibody analysis in the early postacute period between 90 and 160 days after acute infection. Data were recorded using REDCap. Statistical analyses were performed using StataMP 16.1 and 17.0 (StataCorp). We primarily assessed statistical significance by considering the ORs and CIs; a *P* value of less than 0.05 was considered statistically significant.

### Study approval

All participants provided signed written informed consent prior to participation. Institutional Review Board approval was granted by the UCSF.

## Author contributions

MSD designed the study, acquired and analyzed the data, and wrote the first draft of the manuscript. MJP designed the study, acquired the data, provided critical input on data interpretation, and provided critical input on the manuscript. JDK helped with data interpretation and critical edits to the manuscript. SW oversaw echocardiographic measurements and provided critical input to the manuscript. S Swaminathan, DL, VMA, VZ, KS, CH, MIA, RH, and VT acquired data and provided feedback on initial drafts of the manuscript. SL participated in data management and analysis. S Shao participated in acquiring data and designed the graphical abstract. AC, BCY, JWW, and CJP measured biomarkers and antibodies and provided input on the manuscript. JK helped design the statistical plan, data analysis, and interpretation and provided critical edits to the manuscript. TJH, JNM, SGD, and PYH provided critical input on study design, study conduct, and data interpretation and provided edits to the manuscript. MSD had full access to the study data and takes responsibility for the data integrity and analysis.

## Supplementary Material

Supplemental data

## Figures and Tables

**Table 1:**
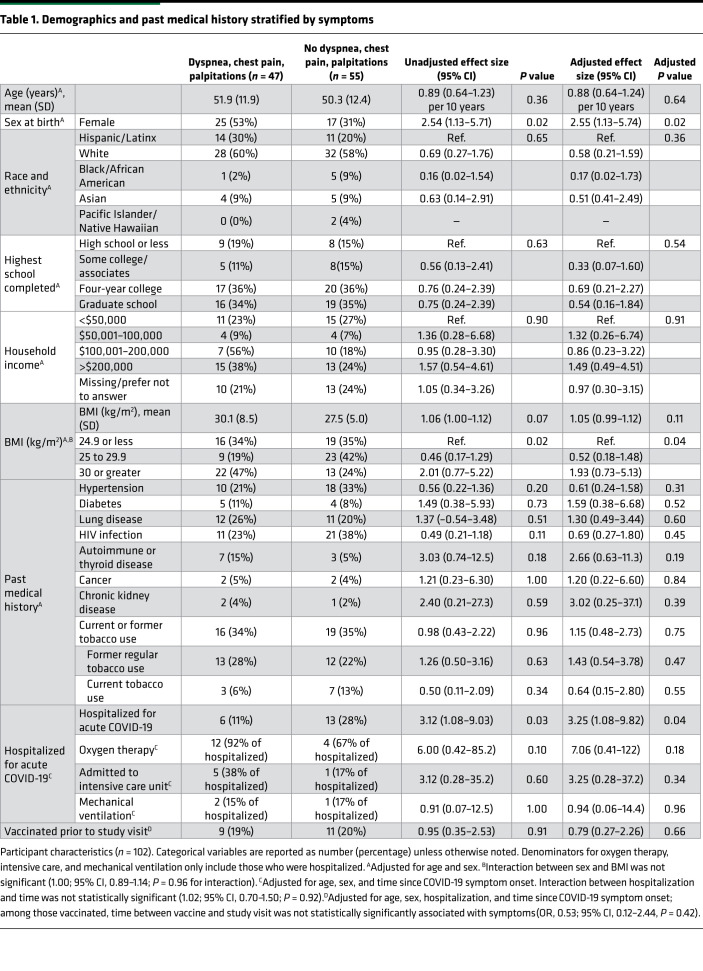
Demographics and past medical history stratified by symptoms

**Table 2:**
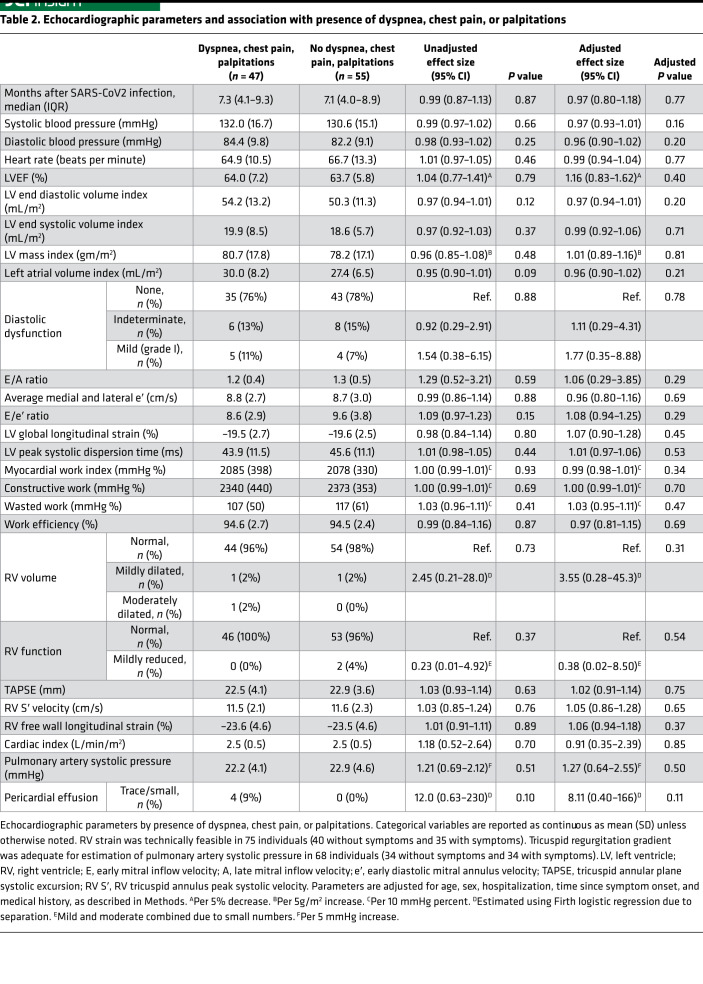
Echocardiographic parameters and association with presence of dyspnea, chest pain, or palpitations

**Table 3:**
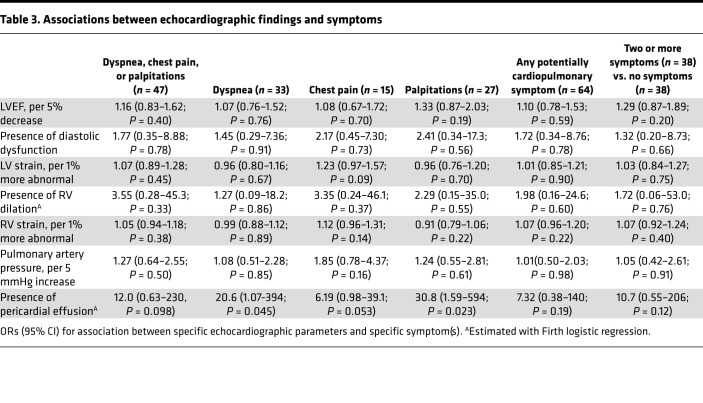
Associations between echocardiographic findings and symptoms

**Table 4:**
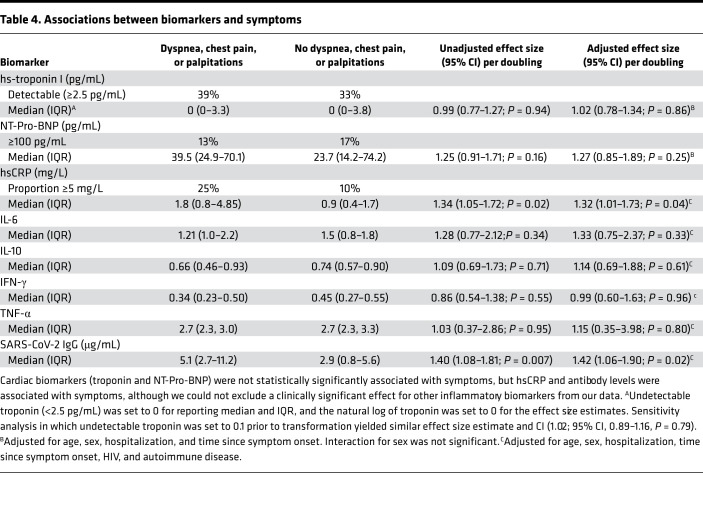
Associations between biomarkers and symptoms

## Version 1 04/07/2022

In-Press Preview

## Version 2 05/23/2022

Electronic publication
