# Supplementary material for: Role of antibodies, inflammatory markers, and echocardiographic findings in postacute cardiopulmonary symptoms after SARS-CoV-2 infection
Source: JCI Insight. 2022 May 23;7(10):e157053. doi: 10.1172/jci.insight.157053 (PMC9220849; doi:10.1172/jci.insight.157053)
Supplement: Supplemental data [file jciinsight-7-157053-s278.pdf]

Supplemental Table 1. Sensitivity Analysis Accounting for Time Since Acute Infection

		Adjusted Effect size (95% CI)	Adjusted p-value	Adjusted effect size including time	New Adjusted P value
Age (years), mean (SD) [a]		0.88 (0.64-1.24) per 10 years	0.64	0.89 (0.63-1.25)	0.49
Sex at Birth[a]	Female	2.55 (1.13-5.74)	0.02	2.55 (1.13-5.74)	0.02
Race/ Ethnicity[a]	Hispanic/Latinx	Ref	0.36	Ref	0.36
	White	0.58 (0.21-1.59)		0.58 (0.21-1.61)	
	Black/African American	0.17 (0.02-1.73)		0.17 (0.02-1.73)	
	Asian	0.51 (0.41-2.49)		0.51 (0.10-2.56)	
	Pacific Islander/ Native Hawaiian	---		---	
Highest School Completed[a]	High school or less	Ref	0.54	Ref	0.54
	Some college/ Associates	0.33 (0.07-1.60)		0.33 (0.07-1.40)	
	4 year college	0.69 (0.21-2.27)		0.69 (0.21-2.28)	
	Graduate school	0.54 (0.16-1.84)		0.53 (0.15-1.84)	
Household Income[a]	<$50,000	Ref	0.91	Ref	0.90
	$50,001-100,000	1.32 (0.26-6.74)		1.30 (0.25-6.72)	
	$100,001-200,000	0.86 (0.23-3.22)		0.87 (0.23-3.31)	
	>$200,000	1.49 (0.49-4.51)		1.51 (0.49-4.63)	
	Missing/Prefer not to answer	0.97 (0.30-3.15)		0.94 (0.27-3.29)	
Body Mass Index (kg/m^2), mean (SD) [a,b]		1.05 (0.99-1.12)	0.11	1.05 (0.99-1.12)	0.11
	24.9 or less	Ref	0.04	Ref	0.04
	25 to 29.9	0.52 (0.18-1.48)		0.51 (0.18-1.47)	
	30 or greater	1.93 (0.73-5.13)		1.93 (0.73-5.13)	
Past Medical History[a]	Hypertension	0.61 (0.24-1.58)	0.31	0.61 (0.23-1.58)	0.31
	Diabetes	1.59 (0.38-6.68)	0.52	1.60 (0.38-6.69)	0.52
	Lung Disease	1.30 (0.49-3.44)	0.60	1.30 (0.49-3.44)	0.60
	HIV Infection	0.69 (0.27-1.80)	0.45	0.69 (0.26-1.80)	0.45
	Autoimmune or Thyroid Disease	2.66 (0.63-11.3)	0.19	2.70 (0.63-11.6)	0.18
	Cancer	1.20 (0.22-6.60)	0.84	1.20 (0.21-6.91)	0.84
	Chronic Kidney Disease	3.02 (0.25-37.1)	0.39	3.12 (0.25-39.1)	0.38
	Current or Former Tobacco Use	1.15 (0.48-2.73)	0.75	1.15 (0.48-2.73)	0.75
Hospitalized for Acute COVID-19[c]		3.25 (1.08-9.82)	0.04		
	Oxygen Therapy [c]	7.06 (0.41-122)	0.18		
	Admitted to Intensive Care Unit [c]	3.25 (0.28-37.2)	0.34		
	Mechanical Ventilation [c]	0.94 (0.06-14.4)	0.96		

Supplemental Table 1 Legend: There were no substantive differences in effect sizes, confidence intervals, or p-values when incorporating time since acute infection in assessing associations between baseline characteristics and symptoms. Additional sensitivity analyses incorporating time as a binary variable (before/after median time), in quartiles, or with use of restricted cubic splines (not shown) did not change the results. Analyses of hospitalization for acute COVID variables already included time since acute infection in the primary analysis as shown in Table 1.

Supplemental Table 2. Sensitivity Analysis Accounting for All Past Medical History

		Adjusted Effect Size (95% CI)	Adjusted p-value	Adjusted Effect Size including all CP Risk Factors	Adjusted p-value
Months after SARS-CoV2 infection, median (IQR)		0.97 (0.80-1.18)	0.77	0.99 (0.84-1.18)	0.92
Vital Signs	Systolic Blood Pressure (mm Hg)	0.97 (0.93-1.01)	0.16	1.00 (0.97-1.03)	0.83
	Diastolic Blood Pressure (mm Hg)	0.96 (0.90-1.02)	0.20	0.98 (0.93-1.03)	0.48
	Heart Rate (beats per minute)	0.99 (0.94-1.04)	0.77	1.01 (0.97-1.06)	0.56
Left Ventricular Parameters	LVEF (%)	1.16 (0.83-1.62)[a]	0.40	1.20 (0.85-1.20)	0.37
	LV End Diastolic Volume Index (ml/m^2)	0.97 (0.94-1.01)	0.20	0.98 (0.94-1.02)	0.32
	LV End Systolic Volume Index (ml/m^2)	0.99 (0.92-1.06)	0.71	0.99 (0.92-1.07)	0.80
	LV Mass Index (gm/m^2)	1.01 (0.89-1.16)[b]	0.81	1.01 (0.87-1.17)	0.89
	Left Atrial Volume Index (ml/m^2)	0.96 (0.90-1.02)	0.21	0.98 (0.92-1.05)	0.58
Diastolic Dysfunction	None	Ref	0.78	Ref	0.56
	Indeterminate	1.11 (0.29-4.31)		1.18 (0.25-5.59)	
	Mild (Grade I)	1.77 (0.35-8.88)		2.69 (0.43-16.9)	
	E/A Ratio	1.06 (0.29-3.85)	0.29	1.07 (0.24-4.73)	0.93
	Average medial & lateral e’ (cm/s)	0.96 (0.80-1.16)	0.69	0.98 (0.78-1.23)	0.85
	E/e’ ratio	1.08 (0.94-1.25)	0.29	1.09 (0.92-1.32)	0.32
Left Ventricular Strain and Myocardial Work	LV Global Longitudinal Strain (%)	1.07 (0.90-1.28)	0.45	1.04 (0.85-1.26)	0.73
	LV peak systolic dispersion time (ms)	1.01 (0.97-1.06)	0.53	1.02 (0.97-1.06)	0.53
	Myocardial Work Index (mm Hg %)	0.99 (0.98-1.01)[c]	0.34	1.00 (0.98-1.01)	0.76
	Constructive Work (mm Hg %)	1.00 (0.99-1.01)[c]	0.70	1.00 (0.99-1.01)	0.92
	Wasted Work (mm Hg %)	1.03 (0.95-1.11)[c]	0.47	1.02 (0.94-1.11)	0.65
	Work Efficiency (%)	0.97 (0.81-1.15)	0.69	1.01 (0.83-1.22)	0.95
RV Volume	Normal	Ref	0.31	Ref	0.44
	Mildly Dilated<br>Moderately Dilated	3.55 (0.28-45.3)[d]		2.60 (0.23-29.1)	
RV Function	Normal	Ref	0.54	Ref	0.64
	Mildly Reduced	0.38 (0.02-8.50)[e]		0.44 (0.01-14.2)	
	TAPSE (mm)	1.02 (0.91-1.14)	0.75	1.03 (0.91-1.17)	0.64
	RV S’ Velocity (cm/s)	1.05 (0.86-1.28)	0.65	1.18 (0.93-1.52)	0.17
RV Strain	RV Free Wall Longitudinal Strain (%)	1.06 (0.94-1.18)	0.37	1.02 (0.89-1.17)	0.75
Hemodynamics	Cardiac Index (L/min/m^2)	0.91 (0.35-2.39)	0.85	0.96 (0.31-2.95)	0.94
	Pulmonary Artery Systolic Pressure (mmHg)	1.27 (0.64-2.55)[f]	0.50	1.51 (0.66-3.45)	0.32
**Pericardial Effusion**	**Trace/ Small**	**8.11 (0.40-166)[d]**	**0.11**	**0.37 (0.32-14.1)**	**0.59**

Supplemental Table 2 Legend: The effect sizes, confidence intervals, and p-values are not substantially different with one notable exception: pericardial effusion. Potential confounders for pericardial effusions include heart failure, renal disease, cirrhosis, thyroid disease, HIV or autoimmune conditions which were not present among any of the individuals with this echo finding. Thus, in our study past medical history seems unlikely to confound the possible association between symptoms and pericardial effusions. As only 4 individuals had pericardial effusions (all symptomatic), adjustment for multiple risk factors is not possible given the small number with this finding.

Supplemental Table 3. Number of potentially cardiopulmonary symptoms among those with and without pericardial effusion

Number of Symptoms	No effusion (n=96)	Pericardial Effusion (n=4)
0	38	0
1	26	0
2	11	0
3	15	1
4	4	3
5	1	0
6	1	0

Supplemental Table 3 Legend: The median number of symptoms among those with a pericardial effusion was 4 compared to 1 among those without pericardial effusion (p=0.0007 by Wilcoxon rank sum). Pericardial effusions were associated with 10.7 times higher odds of having 2 or more symptoms (95%CI 0.55-206; p=0.12).

Supplemental Table 4. Symptoms among whole study sample and those recruited without respect to symptom status

	All participants (n=102)	Participants recruited without respect to symptoms (n=91)	Participants recruited with reported symptoms (n=11)	P value (Fisher's exact)
Chest pain, dyspnea, palpitations	47 (46%)	42 (46%)	5 (45%)	1.00
Chest pain	15 (15%)	13 (14%)	2 (18%)	0.66
Dyspnea	33 (32%)	29 (32%)	4 (36%)	0.74
Palpitations	27 (26%)	22 (24%)	5 (45%)	0.13
Fatigue	32 (32%)	27 (30%)	5 (45%)	0.32
Any symptom (including fatigue)	52 (57%)	52 (57%)	7 (63%)	0.76
2 or more symptoms	38 (37%)	33 (36%)	5 (45%)	0.43

Supplemental Table 4: Number and proportion with the primary outcome, specific symptoms, and 2 or more symptoms based on whether they were recruited based on reported symptoms at a previous LIINC study visit as study enrollment shifted toward symptomatic patients later in the study. Although the proportion with palpitations and fatigue were numerically higher among those recruited later in the study after reporting symptoms, neither was statistically significant (p=0.13 and p=0.32, respectively) and no other symptoms were meaningfully different.

Supplemental Figure 1. Persistence of cardiopulmonary symptoms over time

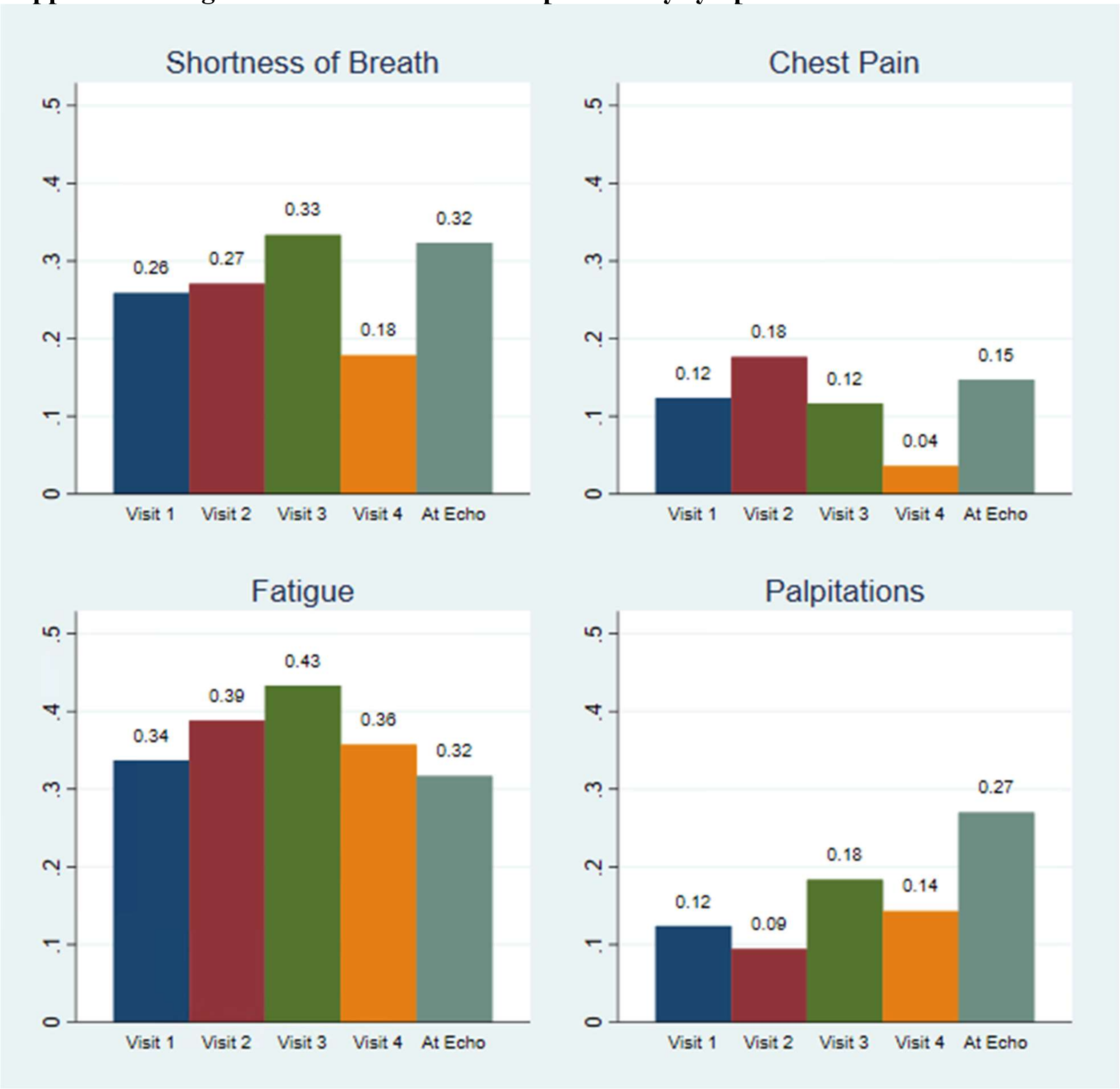

Supplemental Figure 1 Legend: The four panels show the prevalence of shortness of breath, chest pain, fatigue, and palpitations at each study visit; note that these are specific to the study sample and do not represent the true prevalence of these symptoms among all those recovering from COVID-19, but rather demonstrate that the single time point that we present the symptoms is a reasonable approximation to assess the association between biomarkers and echocardiograms. Note that we still adjusted our analyses for time since COVID-19 symptom onset or positive PCR test with the exception of baseline characteristics present at onset of acute infection. Visit 1 took place at a median of 2 months (N=89), Visit 2 at 3 months (N=85), Visit 3 at 4.5 months (N=60), and Visit 4 at 9 months (N=28).

Supplemental Figure 2. Strain and Myocardial Work by Cardiopulmonary Symptoms

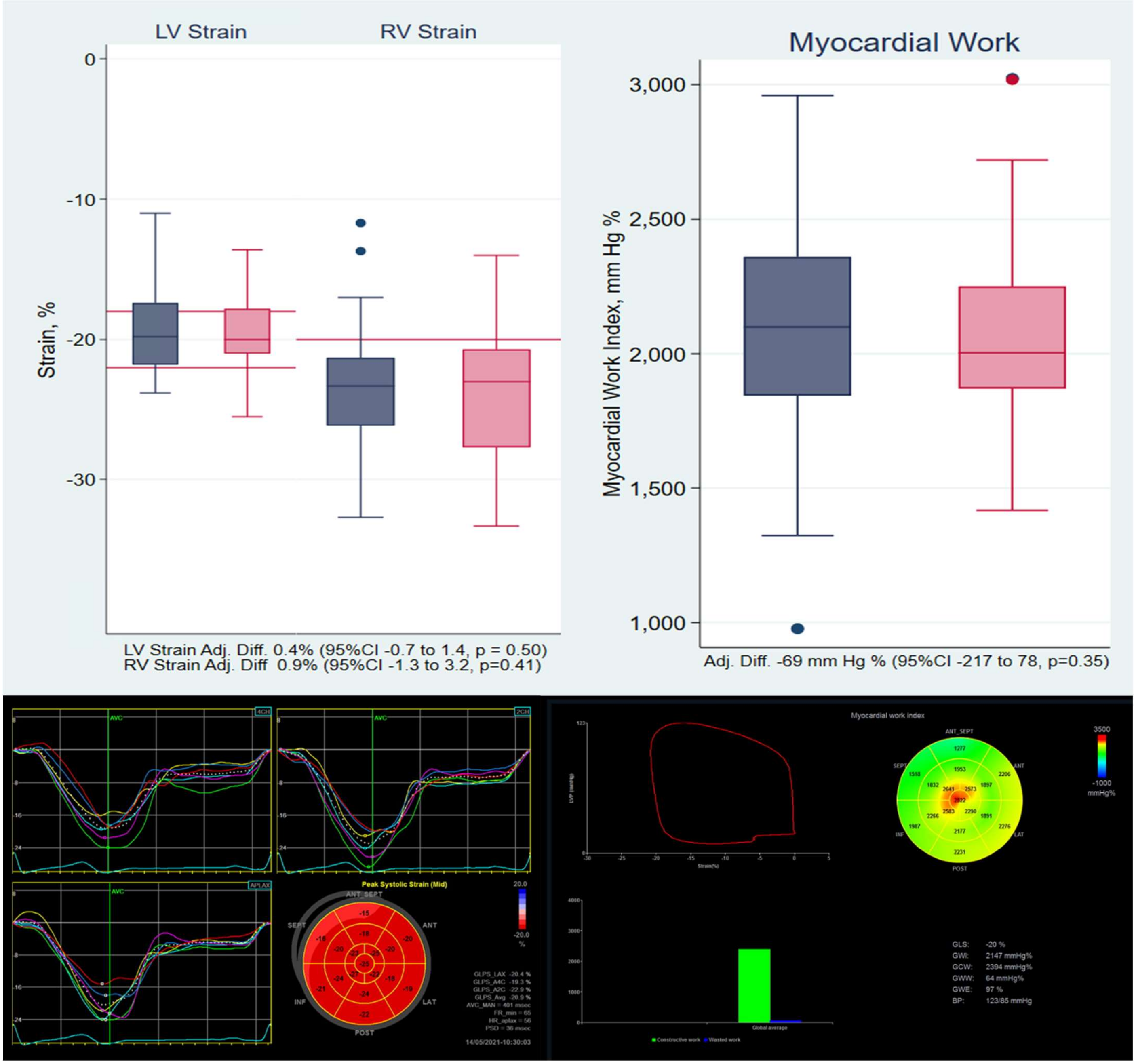

Supplemental Figure 2 Legend: Boxplots of left ventricular peak global longitudinal strain (normal -18 to -22%, red lines),right ventricular strain (normal < -20%, red line), and myocardial work among those without symptoms (navy blue) and with symptoms (pink) are shown on the top. LV strain (OR 1.07, 95%CI 0.89-1.28; p = 0.45), RV strain (OR 1.05, 95%CI 0.94-1.18; p=0.38), and myocardial work (OR 1.00 per 10 mm Hg %, 95%CI 0.99-1.01; p=0.93) were not associated with symptoms. Representative left ventricular strain tracings and myocardial work strain pressure curves and regional myocardial work are shown below.

Supplemental Figure 3. Biomarkers by Presence of Pericardial Effusion

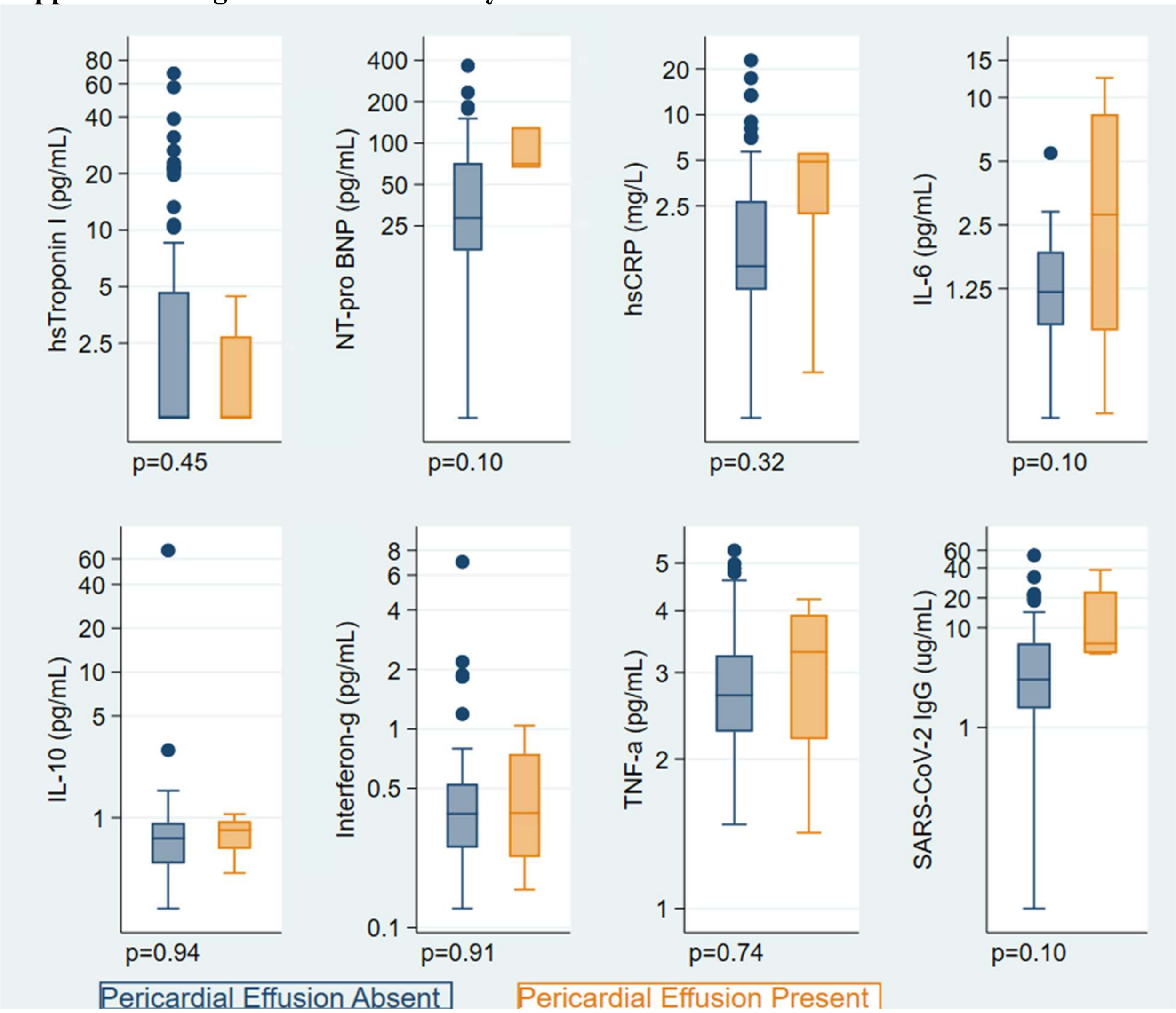

Supplemental Figure 3 Legend. Box and whisker plots of biomarkers by presence of pericardial effusion plotted on log-scale including hs-troponin I, NT-pro-BNP, hs-CRP, IL-6, IL-10, Interferon-gamma, TNF-alpha, and SARS-CoV-2 IgG antibodies. With only 4 individuals with pericardial effusions, the statistical power is low, but our findings suggest higher levels of NT-pro BNP, hsCRP, IL-6, and antibodies may be present among those with pericardial effusions.

Supplemental Figure 4. Relationship between Antibody levels, IL-6, Symptoms and Pericardial Effusions

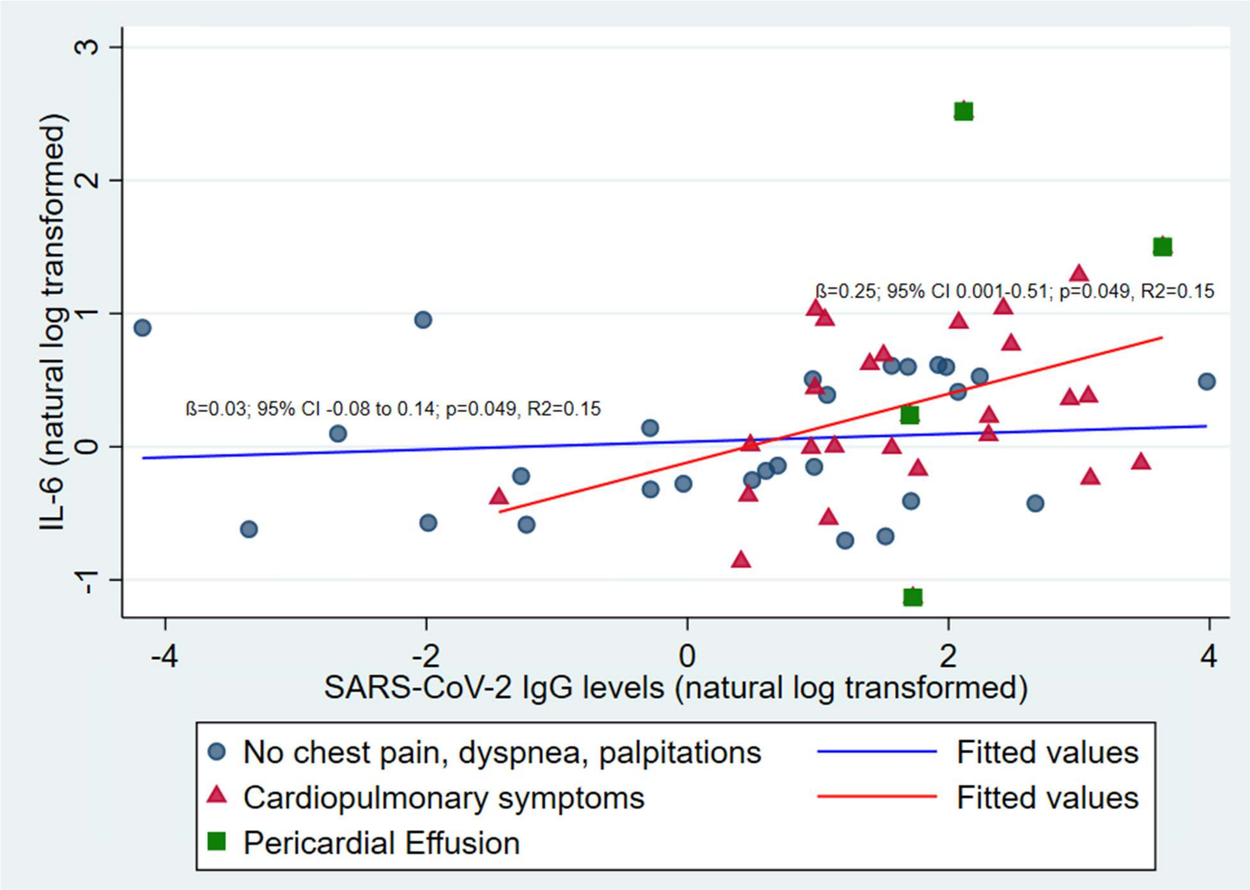

Supplemental Figure 4 Legend: Antibody levels are correlated with IL-6 levels only among those with symptoms (red triangles & trend line; ß=0.25; 95% CI 0.001-0.51; p=0.049, R^2=0.15), but not among those without symptoms (blue circles & trend line; ß=0.03; 95% CI -0.08 to 0.14; p=0.049, R^2=0.15). Individuals with pericardial effusions are shown as green squares.
